# A Comparison of Inhaled Epoprostenol in Patients With Acute Respiratory Distress Syndrome and COVID-19-Associated Acute Respiratory Distress Syndrome

**DOI:** 10.7759/cureus.28274

**Published:** 2022-08-22

**Authors:** John Robinson, Anthony Santarelli, Nathan Wilks, Lelann Latu, Ordessia Charran, Diana Lalitsasivimol, Tyson Dietrich, John Ashurst

**Affiliations:** 1 Pharmacology, Kingman Regional Medical Center, Kingman, USA; 2 Research, Kingman Regional Medical Center, Kingman, USA; 3 Research, Pacific Northwest University of Health Sciences, Yakima, USA; 4 Pulmonary and Critical Care Medicine, Kingman Regional Medical Center, Kingman, USA; 5 Pharmacy / Infectious Diseases, Kingman Regional Medical Center, Kingman, USA; 6 Emergency Medicine, Kingman Regional Medical Center, Kingman, USA

**Keywords:** epoprostenol, cards, ards, pharmacology, covid-19

## Abstract

Introduction

Acute respiratory distress syndrome (ARDS) and coronavirus disease 2019 (COVID-19)-associated acute respiratory distress syndrome (CARDS) are both characterized by non-cardiogenic pulmonary edema and severe hypoxemia that leads to a high percentage of patients suffering in-hospital mortality. Mechanistically, inhaled epoprostenol (iEPO) has shown a role in the treatment of ARDS and CARDS but little data are available directly comparing the two disease processes. Due to the lack of evidence of iEPO in ARDS and CARDS, the authors sought to compare the pulmonary effects of iEPO for mechanically ventilated patients with CARDS against a case match control of those with ARDS.

Methods

A retrospective cohort of all patients receiving iEPO between January 1, 2020, and February 22, 2022, was reviewed. Patients with ARDS were case-matched in a 2:1 allocation ratio of CARDS to ARDS by the number of medical comorbidities and age +/- 5 years. Clinical data collected included patient demographics, laboratory values, ventilator settings, length of hospitalization, and 28-day mortality. Comparisons of the effectiveness of iEPO between ARDS and CARDS were conducted using the chi-squared statistic for categorical variables and the Mann-Whitney statistic for continuous variables.

Results

A total of 72 patients were included in the final analysis, with 24 having ARDS and 48 CARDS. The number of medical comorbidities was no different for patients with ARDS or CARDs (p = 0.18), though the frequency of patients diagnosed with coronary artery disease (p=0.007), congestive heart failure (p=0.003), chronic obstructive pulmonary disease (p=0.004), and pulmonary hypertension (p=0.004) did vary between the two groups. A moderate but non-significant difference in pre-iEPO partial pressure of oxygen (PaO_2_)/fraction of inspired oxygen (FiO_2_) ratio was noted between the groups (0.74 vs 0.65; p=0.33). Following iEPO treatment, patients with ARDS showed a greater PaO_2_/FiO_2_ ratio than those with CARDS (0.87 vs 0.70; p=0.02). CARDS patients who received iEPO had a longer length of stay as compared to those with ARDS (17.5 vs 12.5 days; p=0.01). However, no difference was noted in 28-day mortality between the two groups (14 vs 34; p=0.29).

Conclusion

In this small sample from a single community hospital, a statistically significant improvement in the PaO_2_/FiO_2_ ratio was noted for both those with ARDS and CARDS. However, those with CARDS who were given iEPO had a longer length of stay without a significant difference in mortality as compared to those with traditional ARDS.

## Introduction

Acute respiratory distress syndrome (ARDS) is often a product of noncardiogenic pulmonary edema leading to severe hypoxemia in those who are mechanically ventilated [1]. Due to the increase in patients with ARDS and coronavirus disease 2019 (COVID-19), COVID-19-associated acute respiratory distress syndrome (CARDS) was defined based on the Berlin definition [2,3]. Despite a similar medical definition, those with CARDS show unique pathophysiology post-mortem, including widespread pulmonary vascular thrombi and vascular endothelia accompanied by a lack of the more common ARDS-related alveolar damage [4]. It has been hypothesized that the unique findings in those with CARDS could lead to a greater amount of pulmonary hypertension, right ventricular dysfunction, and an increase in ventilation/perfusion mismatching that is not seen in those with ARDS [4].

Despite varying degrees of evidence and support from national organizations, selective pulmonary vasodilators have been used as a rescue medication to improve oxygenation in those with both refractory ARDS and CARDS [5-12]. Inhaled epoprostenol (iEPO), a synthetic prostacyclin analog, is thought to improve oxygenation through selective pulmonary vasodilatation by cyclic guanosine monophosphate and cyclic adenosine monophosphate-mediated smooth muscle relaxation [5-13]. Previous data have shown iEPO’s ability to alleviate coagulopathy by counteracting the prothrombotic effect of endothelin and decreasing platelet aggregation [14]. Available evidence currently does not show any mortality benefit in those with ARDS or CARDS, but a transient increase in oxygenation has been seen in a subset of both patient populations who have been given iEPO [5-10]. Currently, no data directly compare the outcomes of those with ARDS and CARDS who had received iEPO while mechanically ventilated. As a preliminary investigation, the authors sought to determine if a difference in patient-centered outcomes between those who received iEPO for either ARDS or CARDS at a community hospital existed.

## Materials and methods

Setting

Kingman Regional Medical Center is a 235-bed hospital located in northern Arizona that sees approximately 55,000 patients per year. The facility houses a 14-bed, closed intensive care unit operated by four intensivists.

Protocol

Following Kingman Healthcare Incorporated's institutional review board exemption, a retrospective cohort of all patients who received iEPO between January 01, 2020, and February 22, 2022, was reviewed. Patients were included in the final analysis if they received iEPO for either ARDS or CARDS. Patients who received iEPO for CARDS were then case-matched to those with ARDS.

Inhaled epoprostenol administration

iEPO was administered to patients with either ARDS or CARDS at the discretion of the treating intensivist. When initiated, iEPO was administered via a syringe pump through the inspiratory limb of the mechanical ventilator at an initial dose of 25 nanograms per kilogram of ideal body weight per minute (ng/kg/min). Dosing was then titrated to a maximum of 50 ng/kg/min to induce a clinical response of >10% in PaO_2_ for two hours after initiation.

Case-matching procedure

A total of 24 participants with ARDS were administered iEPO during the study period. Each ARDS patient was used as an index case for the closest available matching to a total of 122 CARDS cases over the same period. The number of comorbidities, including hypertension, diabetes mellitus, chronic kidney disease, coronary artery disease, cancer, congestive heart failure, chronic obstructive pulmonary disease, and pulmonary hypertension, were summed to create a comorbidity load. Patients were then rank-ordered by comorbidity load and age. Patients were then case-matched in a 2:1 allocation ratio of CARDS:ARDS, beginning with the comorbidity load and then age + 5 years.

Data collection

Using the previous definition of response to iEPO, patients were deemed to be an iEPO responder if a 10% increase was noted in the PaO_2_/FiO_2_ ratio between pre and post-arterial blood gases [9,15]. All clinical data reported were abstracted from patient charts from the MEDITECH EXPANSE Platform (Medical Information Technology INC, Westwood, MA). Clinical data collected from the MEDITECH EXPANSE Platform included patient demographics, laboratory values, ventilator settings for a three-day pre/post-initiation period, length of hospitalization, and 28-day all-cause mortality. All data were abstracted by trained research staff who were blinded to the study’s objectives. Research staff were trained on proper data abstraction prior to the collection of data by the study team. With adherence to a quality-controlled protocol and structured abstraction tool, research assistants manually collected all data points, with 20% of all patients being collected in duplicate. Patients with incomplete data or those with inconsistencies were discussed with the investigative team. Patients that had inconsistencies that could not be resolved were removed from the final analysis.

Statistical analysis

The PaO/FiO2 ratio was calculated by using the ratio of arterial oxygen partial pressure to fractional inspired oxygen. Data were analyzed using Statistical Product and Service Solutions (SPSS), v. 27 (IBM Corp., Armonk, NY) with statistical significance being defined as P ≤ 0.05. Categorical variables were assessed for significance via the chi-squared test while continuous variables were assessed via the Mann-Whitney test. An a-priori alpha criterion was set at 0.05 under a two-tailed distribution.

## Results

A total of 72 patients were included in the final analysis, with 24 patients having ARDS and 48 CARDS. In those with ARDS, the median age was 64 (53.3 - 67.6) years with seven (29.2%) being female and having an average of 3.5 (1.3 - 5.8) medical comorbidities (Table 1). In those with CARDS, the median age was 65.0 (52.5 - 71.8) years with 17 (35.4%) being female and having an average of 3.0 (1.3 - 4.0) medical comorbidities. Those in the ARDS group were more likely to have coronary artery disease (33.3% vs 8.3%; p=0.007), congestive heart failure (50.0% vs 16.7%; p=0.003), chronic obstructive pulmonary disease (54.2% vs 20.8%; p=0.004), and pulmonary hypertension (29.2% vs 10.4%; p=0.04) than those in the matched CARDS group.

**Table 1:** Baseline demographics of those with acute respiratory distress syndrome and COVID-19 acute respiratory distress syndrome ARDS: acute respiratory distress syndrome; CARDS: COVID-19 acute respiratory distress syndrome; COVID-19: coronavirus disease 2019

Characteristic	ARDS (N = 24)	CARDS (N = 48)	P-Value
Age	64.0 (53.3 – 67.5)	65.0 (52.5 – 71.8)	0.31
Female	7 (29.2%)	17 (35.4%)	0.60
Hypertension	16 (66.7%)	34 (70.8%)	0.72
Diabetes	12 (50.0%)	27 (56.3%)	0.62
Chronic kidney disease	6 (25.0%)	7 (14.6%)	0.28
Coronary artery disease	8 (33.3%)	4 (8.3%)	0.007
Cancer	1 (4.2%)	6 (12.5%)	0.26
Congestive heart failure	12 (50.0%)	8 (16.7%)	0.003
Chronic obstructive pulmonary disease	13 (54.2%)	10 (20.8%)	0.004
Pulmonary hypertension	7 (29.2%)	5 (10.4%)	0.04
Number of comorbidities	3.5 (1.3 – 5.8)	3.0 (1.3 – 4.0)	0.18

No difference was noted in the PaO_2_/FiO_2_ ratio in those with ARDS and CARDS prior to iEPO treatment (0.74 vs 0.65; p=0.33) (Table 2). Following treatment with iEPO, the PaO_2_/FiO_2_ ratio of those with ARDS was higher than that in those with CARDS (0.87 vs 0.70; p=0.02). Though not statistically significant, a higher percentage of patients with ARDs (58.3%, 14/24) showed a positive clinical response to iEPO of a 10% increase in PaO_2_/FiO_2_ than those in those with CARDs (41.7%, 20/48) (p = 0.182). Patients with CARDS who received iEPO had a longer length of hospitalization than those with ARDS (17.5 vs 12.5 days; p=0.01). No difference in 28-day mortality was noted between the two groups (14 vs 34 patients; p=0.29).

**Table 2:** Patient-centered outcomes for those with acute respiratory distress syndrome and COVID-19 acute respiratory distress syndrome who received inhaled epoprostenol ARDS: acute respiratory distress syndrome; CARDS: COVID-19 acute respiratory distress syndrome; COVID-19: coronavirus disease 2019; iEPO: inhaled epoprostenol; PaO2: partial pressure of oxygen; FiO2: fraction of inspired oxygen

	ARDS (N=24)	CARDS (N=48)	P-Value
Pre PaO2/FiO2, mmHg	0.74 (0.54 – 1.05)	0.65 (0.53 – 0.88)	0.33
Post PaO2/FiO2, mmHg	0.87 (0.71 – 1.48)	0.70 (0.59 – 1.0)	0.02
Post PaO2/FiO2, mmHg; Pre PaO2/FiO2, mmHg	1.19 (0.96 – 1.59)	1.05 (0.94 – 1.30)	0.152
> 10% Post-iEPO PaO2/FiO2	58.3% (14/24)	41.7% (20/48)	0.182
Length of stay	12.5 (7.3 – 20.5)	17.5 (14.0 – 30.8_	0.01
28-day mortality	14 (58.3%)	34 (70.8%)	0.29

No difference in the pre PaO_2_/FiO_2_ ratio was noted for males who had either ARDS or CARDS (0.74 vs 0.64; p=0.20) (Table 3). Following iEPO therapy, males saw an increase in their PaO_2_/FiO_2_ ratio with those who had ARDS being larger than those with CARDS (1.04 vs 0.67; p=0.01). After iEPO therapy, males with CARDS stayed in the hospital longer than those with ARDS (17 vs 12 days; p=0.04) but no difference in 28-day mortality was noted between the two groups (24 vs 9 patients; p=0.08). When females with ARDS and CARDS were considered, no difference in the pre-PaO2/FiO2 ratio, post-PaO2/FiO2 ratio, length of stay, and 28-day mortality was noted after iEPO therapy (Table 3).

**Table 3:** Patient-centered outcomes based upon sex for those with acute respiratory distress syndrome and COVID-19 acute respiratory distress syndrome who received inhaled epoprostenol ARDS: acute respiratory distress syndrome; CARDS: COVID-19 acute respiratory distress syndrome; COVID-19: coronavirus disease 2019; PaO2: partial pressure of oxygen; FiO2: fraction of inspired oxygen

	ARDS Male (N = 17)	CARDS Male (N = 31)	P-Value		ARDS Female (N = 7)	CARDS Female (N = 17)	P-Value
Pre PaO_2_/FiO_2_, mmHg	0.74 (0.54 – 1.09)	0.64 (0.53 – 0.87)	0.20		0.68 (0.53 – 0.91)	0.65 (0.54 – 0.98)	0.95
Post PaO_2_/FiO_2_, mmHg	1.04 (0.74 – 1.0)	0.67 (0.59 – 0.94)	0.01		0.83 (0.59 – 1.21)	0.82 (0.57 – 1.07)	0.80
Length of stay	12.0 (8.0 – 20.5)	17.0 (14.0 – 29.0)	0.04		13.0 (4.0 – 21.0)	18.0 (13.0 – 33.5)	0.17
28-day mortality	9 (52.9%)	24 (77.4%)	0.08		5 (71.4%)	10 (58.8)	0.56

In total, six patients with ARDS and 16 with CARDS were placed into the prone position following initiation of iEPO. No difference in the Pre PaO_2_/FiO_2_ ratio was noted between the groups (1.0 vs 0.65; p=0.13) (Table 4). Both groups saw an increase in the post-PaO2/FiO_2_ ratio with those with ARDS having a post-PaO_2_/FiO_2_ ratio that was larger than those with CARDS (1.13 vs 0.75, p=0.04). No difference between those with ARDS and CARDS was noted in the length of stay (12 vs 15.5 days; p=0.18) or 28-day mortality (5 vs 12 patients; p=0.68) when they were given iEPO and were placed into the prone position.

**Table 4:** Patient-centered outcomes for those who were placed in the prone position and received inhaled epoprostenol with acute respiratory distress syndrome and COVID-19 acute respiratory distress syndrome ARDS: acute respiratory distress syndrome; CARDS: COVID-19 acute respiratory distress syndrome; PaO2: partial pressure of oxygen; FiO2: fraction of inspired oxygen

	ARDS Prone (N = 6)	CARDS Prone (N = 16)	P-Value
Pre PaO_2_/FiO_2_, mmHg	1.0 (0.55 – 1.21)	0.65 (0.48 – 0.88)	0.13
Post PaO_2_/FiO_2_, mmHg	1.13 (0.81 – 1.55)	0.75 (0.85 – 0.75)	0.04
Length of stay	12.0 (7.8 – 14.8)	15.5 (10.5 – 23.8)	0.18
28-day mortality	5 (83.3%)	12 (75.0%)	0.68

## Discussion

Currently, no randomized control trials have examined the effects of iEPO when used for the treatment of CARDS and no studies have compared its usage between those with ARDS and CARDS. To begin to describe the efficacy of iEPO for each condition, the authors sought to complete a preliminary assessment in a retrospective cohort. Much like previous data on both ARDS and CARDS, an improvement in the PaO_2_/FiO_2_ ratio was noted in both groups after the administration of iEPO [5-10]. Mechanistically, this is most likely due to a multitude of effects that include vascular smooth muscle relaxation, pulmonary vasodilation, and platelet stabilization [4]. However, those with ARDS had a greater post PaO_2_/FiO_2 _ratio as compared to those with CARDS. This could be due to the differences in the pathophysiology seen in those with CARDS as compared to traditional ARDS or the usage of iEPO as rescue therapy in patients with severe hypoxemia due to COVID-19 [2]. Due to the magnitude of effect difference between ARDS and CARDS patients in the cohort, this data suggests a more rigorous evaluation of dosing procedures for iEPO when administered to patients with CARDS is needed.

Despite these improvements in the PaO_2_/FiO_2_ ratios seen, no difference in 28-day mortality was noted between the two groups. Instead, CARDS patients who were given iEPO had a longer length of hospitalization prior to succumbing to their illness. It is unclear the exact reason for those with CARDS having an increased length of hospitalization but is most likely related to a multifactorial process involving the progression of the disease and the timing of a patient’s illness. These data are consistent with recently published data that showed that those with CARDS had a 6.5-day longer stay than those with traditional ARDS [16]. Prior to making a formal recommendation on the usage of iEPO in CARDS, however, further randomized controlled trials are needed to assess the effects on patient-centered outcomes, including elucidating the reasons why patients with CARDS had a longer LOS as compared to those with ARDS.

In this sample, males with CARDS had a smaller improvement in their post PaO_2_/FiO_2_ ratios as compared to males with ARDS or females with ARDS or CARDS. Previous data in CARDS has shown a varying degree of responsiveness to iEPO among a cohort of mechanically ventilated individuals [7,9,10]. However, none of these studies has assessed the response rate between the biological sexes [7,9,10]. These findings are interesting and serve as a potential for future research to determine why these sex-specific differences were found since responsiveness to iEPO in both ARDS and CARDS should be related to the degree of ventilation/perfusion mismatching of each individual patient. Further data are needed to determine the effectiveness of iEPO between the different biological sexes in both ARDS and CARDS.

Prior to the COVID-19 pandemic, placing patients with severe ARDS in the prone position was shown to improve oxygenation and reduce 28 and 90-day mortality [15,17]. During the pandemic, the Surviving Sepsis Campaign COVID-19 subcommittee made a similar recommendation to place those with severe hypoxemia and COVID-19 into the prone position as a means of decreasing ventilation/perfusion mismatching [11]. In this limited sample, both those with ARDS and CARDS showed a clinical improvement in their pre/post PaO_2_/FiO_2_ ratios. This improvement has been theorized to be related to a more homogenous distribution of iEPO due to the improvement in ventilation/perfusion mismatching [4]. Although a statistically significant difference in the PaO_2_/FiO_2_ ratios was noted, no difference in the length of hospitalization or 28-day mortality was seen when the two groups were compared. This could be due to both the relatively low sample size and examining the entire cohort against one another instead of those who were deemed responders versus non-responders to iEPO therapy.

Limitations

Cases were matched based on sex and the number of medical comorbidities, other confounding variables that were not accounted for may have influenced the results. The authors did not match patients based upon 10-year survivability (Charlson Comorbidity Index) or ICU survivability (APACHE II score). If using a different method for matching, the final results may have been different than what was currently found. Although the hospital had a formalized protocol for treatment, the initiation of the protocol was variable amongst intensivists. Treatment decisions, including that of placing a patient into the prone position, were at the sole discretion of the treating physician. Data may also not be generalizable to all facilities due to the geographical location and relatively homogenous patient population seen by the study facility. The results are limited due to the small sample size and further larger scale or multisite research may be needed to truly elucidate the treatment response of those given iEPO in those with ARDS and CARDS.

## Conclusions

At a single community hospital in northern Arizona, iEPO shows clinical utility to improve the PaO_2_/FiO_2_ ratio in those with both ARDS and CARDS. In those with CARDS, however, patients had a greater length of hospitalization following treatment but no difference in overall mortality at 28 days as compared to ARDS. This finding is likely confounded by a multitude of variables in both treatment strategies and the progression of illness between patients. Given the study’s small sample size, further randomized trials are needed before any definitive statements are made on the effects of iEPO in CARDS as compared to ARDS.
